# The possibilities of using elastic therapeutic (Kinesio) tape in patients with scoliosis

**DOI:** 10.1186/1748-7161-9-S1-P12

**Published:** 2014-12-04

**Authors:** Esther de Ru

**Affiliations:** 1 GoPhysio, Zutphen, Netherlands

## 

Elastic Therapeutic (Kinesio Tape) has rapidly become a recognised therapeutic modality in many musculoskeletal and neurological disorders. This tape, applied with a certain amount of stretch to its own paper backing is known under many names making research into it a challenging task. Its recoil properties make it a very suitable tool to assist in current scoliosis management.

The studies of the various elastic therapeutic tape brands have reported positive effects on pain, ROM, balance, strength, function and proprioception. A large amount of anecdotal evidence has been published and warrants further research. It is reasonable to assume that the outcome of the research on the effects of taping on both upper and lower extremities by means of Functional Magnetic Resonance Imaging and Ultrasonic Imaging is transferable to the trunk. Both the research on the physiological movement of the skin of the trunk captured by three-dimensional motion analysis system (Vicon Motion Systems) by T. Fukui and the videos of the skins’ anatomy and physiology by Dr. J.C. Guimberteau have resulted in novel ways to use the tape.

A number of these tape possibilities will be presented and their function addressed.

The use of elastic therapeutic tape applications to:

- assist patients to 'hold' their corrected posture (Schroth, SEAS exercises). In both Idiopathic Adolescent Scoliosis and adult scoliosis patients this extra stimulus gives 24hours a day sensory information.

- help relieve pain in the (elderly) scoliosis patient with postural collapse.

- assist pulmonary function in the both patients with Idiopathic Adolescent Scoliosis and neuromuscular scoliosis will all be presented and discussed.

Elastic Therapeutic Tape is a wonderful new extra tool in the management of scoliosis patients.

